# Volumetric reconstruction from printed films: Enabling 30 year longitudinal analysis in MR neuroimaging

**DOI:** 10.1016/j.neuroimage.2017.09.056

**Published:** 2018-01-15

**Authors:** Michael Ebner, Karen K. Chung, Ferran Prados, M. Jorge Cardoso, Declan T. Chard, Tom Vercauteren, Sébastien Ourselin

**Affiliations:** a Translational Imaging Group (TIG), Centre for Medical Image Computing (CMIC), Department of Medical Physics and Biomedical Engineering, University College London (UCL), London, UK; b Nuclear Magnetic Resonance (NMR) Research Unit, Queen Square Multiple Sclerosis (MS) Centre, Department of Neuroinflammation, UCL Institute of Neurology, London, UK; c National Institute for Health Research (NIHR) University College London Hospitals (UCLH) Biomedical Research Centre (BRC), London, UK; d Wellcome / EPSRC Centre for Interventional and Surgical Sciences (WEISS), University College London, London, UK

**Keywords:** Historical MR film data, Brain MRI, Regularized image registration, Total variation reconstruction, Longitudinal analysis

## Abstract

Hospitals often hold historical MR image data printed on films without being able to make it accessible to modern image processing techniques. Having the possibility to recover geometrically consistent, volumetric images from scans acquired decades ago will enable more comprehensive, longitudinal studies to understand disease progressions. In this paper, we propose a consistent framework to reconstruct a volumetric representation from printed films holding thick single-slice brain MR image acquisitions dating back to the 1980's. We introduce a flexible framework based on semi-automatic slice extraction, followed by automated slice-to-volume registration with inter-slice transformation regularisation and slice intensity correction. Our algorithm is robust against numerous detrimental effects being present in archaic films. A subsequent, isotropic total variation deconvolution technique revitalises the visual appearance of the obtained volumes. We assess the accuracy and perform the validation of our reconstruction framework on a uniquely long-term MRI dataset where a ground-truth is available. This method will be used to facilitate a robust longitudinal analysis spanning 30 years of MRI scans.

## Graphical abstract

**Figure:**
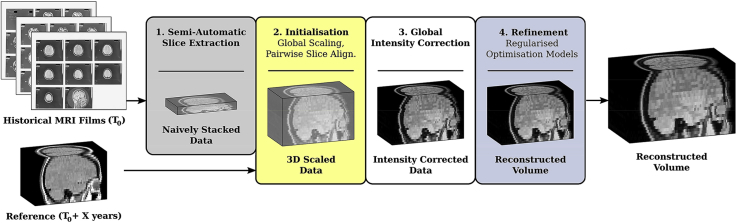

## 1 Introduction

**Fig. 1:**
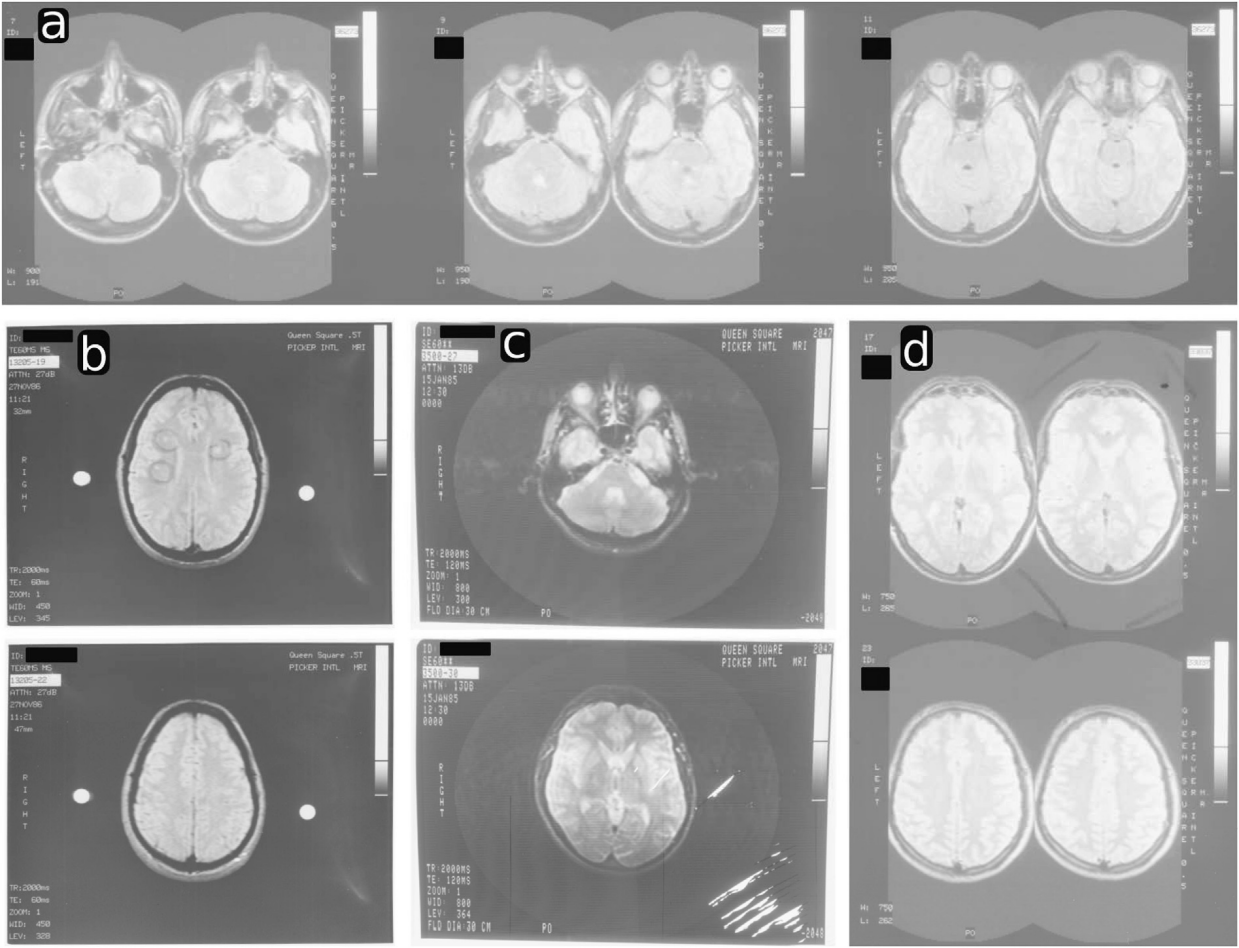
Scanned MR Films of MS/Clinically Isolated Syndrome (CIS) subjects from 1985 to 1991. The top row (a) illustrates a typical proton density-like sequence of printed 5 mm thick slice acquisitions side-by-side, acquired at 1.5T in 1991. The left bottom column (b) illustrates a scan acquired at 0.5T in 1986 with lesions encircled directly on the film. The middle bottom column (c) depicts a scratched, visibly rotated and deformed film from 1985 with unknown slice-thickness. The bottom right column (d) shows handwritten annotations on a scan from 1990. The skulls and also a part of the brain is merged into each other to save film space. Overall, it is worth noting the different types of MR films, their illumination differences and different visible distortions affecting even the same film belonging to the same acquisition.

Since the early 1980's, when it first became available for clinical use, Magnetic Resonance Imaging (MRI) has been recognised as a powerful, non-invasive and non-ionising medical imaging technique (Damadian, 1971). The earliest, longitudinal brain studies were performed based on thick contiguous slices acquired in the axial direction to cover the entire volume, e.g. (Miller et al., 1988, Miller et al., 1989). In absence of modern standards for digital archives and visualisation, the acquired scans were placed side-by-side and printed sequentially on multiple films, for further, *visual* analysis, as shown in Fig. 1. The analysis was typically limited to measures such as lesion count and location in multiple sclerosis (MS) studies (Miller et al., 1989, Morrissey et al., 1993, O'Riordan et al., 1998). With the introduction of the PACS (Picture Archiving and Communications System) and DICOM (Digital Imaging and COmmunication in Medicine) standards in the beginning of the 90's, the basis was created to digitally store medical imaging information including essential meta-data on spatial information and acquisition details. This allowed further development of clinically important biomarkers such as brain and lesion volume for longitudinal MS studies (Brex et al., 2002, De Stefano et al., 2014, Sailer et al., 1999) – information which, currently, cannot be readily extracted from scans dating back to the 80's and early 90's if they are only available as printed films. The original, digital data is often lost or cannot be recovered due to hardware and software obsolescence issues which has also been shown in more recent studies on brain morphometry where original MR films were digitised anew and manually processed to allow for further quantification (Ekert et al., 2016). In other words, especially for longitudinal studies dating back to the 80's, *a decade or more of valuable image data information may not be readily accessible to modern image processing techniques* which could add to the understanding of long-term pathological or morphological evolution.

In order to re-establish a consistent, volumetric representation from printed, historical films several challenges need to be overcome: Patient-specific anatomy is only sparsely captured on printed films corresponding to a single acquisition of axially acquired thick slices only. Each 2D slice needs to be extracted from the MR film and correctly aligned in the 3D space. Slice-based motion correction techniques have been successfully applied in various fields of medical imaging (Ferrante and Paragios, 2017) and can estimate the correct inter-spatial relationship of multiple slices to reconstruct a consistent volumetric representation. Applications of slice-based motion correction and reconstruction methods in MRI include the challenging problems of fetal MRI (Kainz et al., 2015a, Kim et al., 2010b, Rousseau et al., 2006, Tourbier et al., 2015) or abdominal MRI (Ebner et al., 2017) in structural imaging but also functional MRI (Kim et al., 1999, Seshamani et al., 2016) and diffusion tensor imaging (DTI) (Jiang et al., 2009, Fogtmann et al., 2014, Marami et al., 2016, Marami et al., 2017). In general, the lower the slice resolution, the more ill-posed the registration and reconstruction problems become. Therefore, *multiple* stacks of slices in single (Jiang et al., 2009, Kim et al., 1999, Seshamani et al., 2016) or multiple (Ebner et al., 2017, Fogtmann et al., 2012, Fogtmann et al., 2014, Marami et al., 2017, Kainz et al., 2015a, Kim et al., 2010b, Rousseau et al., 2006, Tourbier et al., 2015) imaging planes are typically acquired in order to obtain a sufficiently dense data sampling to better constrain the motion correction and reconstruction problems. In particular, Fogtmann et al., 2012, Fogtmann et al., 2014 and, more recently, Marami et al. (2017) have proposed regularised slice-to-volume registration approaches to better address the ill-posed nature of independent slice-to-volume registrations in order to achieve more robust motion correction frameworks for multi-plane multi-slice Diffusion MRI. In the case of reconstructing volumetric representations from printed MR films, however, only one *single* stack of past thick-slice acquisitions in a *single, axial plane* is captured. The slice thickness of past acquisitions can range from 5 mm like in Brex et al., 2002, Ekert et al., 2016, Miller et al., 1989, Sailer et al., 1999 to 10 mm as in Brex et al., 2002, Miller et al., 1989, Sailer et al., 1999 to even encountered 12 mm. Hence, even in the 5 mm slice thickness case, which is the focus of this work, neighbouring slices correspond to relatively distant anatomical positions which renders purely intra-stack alignment-based motion correction approaches particularly difficult so as to recover the correct inter-slice relationship and, thus, the patient-specific anatomy. An appropriate single slice-based motion correction approach will be key but needs to deal with the very sparse information given the thick, contiguous slices. Additionally, the geometrical properties and dimensions of printed slices are lost and need to be recovered. The top part of the brain is often missing due to a reduced field of view (FOV) in past acquisitions complicating accurate registrations. The arguably higher magnetic field inhomogeneities of past MR image acquisitions and the further processing associated with film printing, storage and subsequent scanning lead to different types of illumination differences which are present across, but also within, MR films belonging to the same acquisition in addition to other types of degradations as shown in Fig. 1. Storage of the films over decades may have further degraded the data whereby individual films belonging to the same acquisition may have been affected differently resulting in stark differences in image intensities across slices of different films. Moreover, historical films are likely to carry a substantial amount of background noise and may well come with low image contrast. Additional distortion has been introduced due to the performed manual scanning, manifested in rotated, sheared and possibly, otherwise deformed images, as visible in Fig. 1. Due to advances in MR, increased field strengths, higher spatial resolution, changes in imaging protocols and image contrast preferences for diagnostic purposes in addition to changes in MR scanner manufacturers and printers, the appearance and also the layout of MR films can change substantially in the course of a longitudinal study spanning several decades.

In this paper, we propose a novel reconstruction framework, able to address the challenges discussed above. More specifically, our contributions are:

1. A semi-automatic slice extraction tool to create a digital image stack from historical slices selected from the scanned brain MR films. It provides an initial digital 3D representation of acquired slices printed on a 2D film where the correct spatial position and dimension of each single slice needs to be recovered.
2. A fully automatic volumetric reconstruction framework to estimate the lost meta-data information of each slice in the 3D space. It is based on a joint slice-to-volume affine registration with inter-slice 2D transformation regularisation and affine slice-intensity correction. Missing meta-data information is contributed by a longitudinal scan of the same subject. A final isotropic total variation in-plane deconvolution technique serves to revitalise the visual appearance of the reconstructed stack of historical slices.
3. A validation of our slice-extraction tool and volumetric reconstruction framework on clinical, *historical ground-truth* data to show the potential of our proposed framework to enable a more robust analysis of long-term datasets:
  • We apply our proposed method to a uniquely long-term, longitudinal dataset of patients first recruited with clinically isolated syndrome (CIS) dating back to the 1980's (Miller et al., 1988, Miller et al., 1989, Morrissey et al., 1993, O'Riordan et al., 1998, Brex et al., 2002, Fisniku et al., 2008).
  • We validate our framework on a subset of this cohort where also the original, digital stack of the same acquisition has been preserved in addition to the printed MR films. In this rare situation, we can validate against *historical ground-truth* data.
    – We perform a quantitative comparison and assess the accuracy of our obtained volumetric reconstructions in terms of mean squared error, normalised cross correlation, structural similarity, peak signal-to-noise ratio and Structural Image Evaluation, using Normalization, of Atrophy (SIENA) (Smith et al., 2002) analysis.
    – We undertake a qualitative assessment relying on expert neurologist ratings both in terms of clinical usefulness and ground-truth comparison of our recovered volumetric representations of historical film data.
    – We provide a qualitative comparison of longitudinal data to assess ground-truth similarity over time.

The framework is made open source and available on github.^1^

Compared to regularised slice-to-volume motion-correction and MR reconstruction methods proposed in the literature such as (Fogtmann et al., 2012, Fogtmann et al., 2014) or (Marami et al., 2017) the proposed reconstruction pipeline differs significantly in a number of aspects as it is designed particularly to deal with the specific challenges associated with the volumetric reconstruction from historical MR films.

Fogtmann et al. (2014) propose reconstructing 3D DTI from multiple multi-slice diffusion weighted (DW) images by using a framework for unified motion-estimation and image reconstruction as an extension of their previous work (Fogtmann et al., 2012) introduced for structural multi-plane MRI. Despite the formulation as a unified approach, the volumetric reconstruction of the unknown image is performed by alternating between the two problems of estimating the motion parameters of a 3D rigid and scale-skewness transform for all slices followed by estimating the weight parameters which define the diffusion volume. Instead, we propose a method which corrects at once for affine in-plane 2D motion of each single slice and estimates its missing physical dimensions by the guidance of a reference volume acquired many years later which usually exists in longitudinal studies. In particular, this approach avoids out-of-plane resampling of the very sparse anatomical data given by only one single stack of the thick axial slices. Moreover, Fogtmann et al. (2014) use a regularisation prior based on the Huber norm for motion correction to favour similarity between motion correction parameters of consecutive slices. We propose a robust smooth $\ell^{1}$-approximation-based inter-slice affine 2D transform regularisation and affine intensity correction framework in addition to the use of a prior on optimisation parameters based on a smooth $\ell^{1}$-approximation. This drives the physical dimension and 2D position estimates directly by the similarity between slice neighbours guided by the anatomical shape prior provided by the reference volume.

Marami et al. (2017) build on their work presented in (Marami et al., 2016) and explicitly model the dynamics of rigid motion with a state space model where they estimate the temporal motion trajectories with a Kalman filter for a more robust reconstruction of DWI. They automatically detect and reject motion-corrupted DWI slices to enhance motion tracking and reconstruction. In our setting of volumetrically reconstructing 3D volumes from sparse historical 2D slices printed on films, the motion captured in the obtained stack of slices after the semi-automatic slice extraction cannot be assumed to follow a physiological model. Each individual slice will have different motion shifts with respect to each other given that each slice is extracted according to a landmark which is placed manually on the film. Therefore, we propose a motion correction framework based on an inter-slice regularisation which leverages the 2D image similarity between two neighbour slices and the respective (oblique) reference slice instead. Moreover, we do not perform outlier rejection as we need to recover the physical position and dimension for each single slice reliably in order to form a consistent volumetric representation of the subject-specific anatomy as captured by the single acquisition in the past. Importantly, however, they use the method presented in (Kainz et al., 2015b) for the required structural image reconstruction of the high-resolution T2-weighted volume from multiple motion corrupted slices, which uses independent rigid slice-to-volume registration without regularisation.

In addition, the mentioned approaches rely on Super-Resolution techniques (Park et al., 2003, Gholipour et al., 2010) for the image reconstruction steps to reconstruct a single, higher-resolution, isotropic 3D volume from multiple scattered low-resolution 2D slices. In our approach, a final isotropic total variation in-plane deconvolution step is added after performed in-plane motion correction of each single slice for improved visual appearance only.

The remaining part of this paper is organised as follows. Section 2 motivates and presents the design choice and the details of our proposed volumetric reconstruction framework. In Section 3 the results of the validation of our proposed volumetric reconstruction framework are presented using a uniquely long-term historical dataset spanning 30 years of MRI scans. Finally, Section 4 concludes with a discussion.

## 2 Volumetric reconstruction from printed MR films

The first step of the volumetric reconstruction method is dedicated to the slice extraction and stacking of all slices of the same historical axial acquisition in order to create an initial, digital 3D image. A semi-automatic slice extraction framework is chosen to deal with the wide variety of existing films, data characteristics, and styles as pointed out in Fig. 1. This enables flexible processing even for complex cases where slices are merged on a printout and brain images need to be carefully delineated, as, e.g., shown in Fig. 1a. A manual interaction can additionally ensure that only correct slices and films belonging to the same acquisition are extracted. This is particularly relevant since multiple films, or slices printed on films, encountered in the database of historical films are duplicated whereas other ones are missing or not ordered in the right sequence and inevitably require manual intervention.

The imperfect slice extraction of rotated, sheared and possibly otherwise deformed images due to printing and manual scanning gives rise to a naively stacked 3D data with visibly in-plane motion affected slices. Therefore, the volumetric reconstruction framework needs to recover the correct inter-spatial position of all slices in addition to their physical dimensions. After printing and scanning, the only spatial information available from the MR films is the slice thickness which is generally printed on the films as indicated in Fig. 1. All other meta-data typically stored in DICOM headers, such as exact spatial position relative to the neighbouring slice, in-plane spacing, and image orientation, is lost and needs to be recovered for each single slice. The aim of our algorithm is to infer the missing information from a more recent, digital 3D scan of the same patient which holds the required meta-data information and is of similar intensity contrast; a scan which generally exists in longitudinal studies. For instance, early and current studies for MS use proton density (PD)-like image contrast (Gracien et al., 2016, Miller et al., 1988) despite the advances and changes in imaging protocols over several decades. However, this reference image is likely to be acquired many years later and the patient may have undergone substantial morphological changes including atrophy. It will be dissimilar to the brain captured by the historical MR films.

By neglecting subject motion during acquisition time, slice motion correction can be reduced to in-plane motion correction only. This assumption addresses both the need of balancing the complexity versus robustness of the method and avoids out-of-plane resampling for the final volumetric reconstruction of the sparse, historical, thick-sliced data. The primary goal of our obtained volumetric reconstructions from printed films is to gain clinical trust by performing a sufficiently accurate motion correction without introducing implausible deformations. Hence, a gradual increase of transformation complexity shall be performed up to in-plane 2D affine transformation which is believed to be capable of dealing with the encountered distortions in the films. Intra-stack slice registration is highly ill-posed due to the thick slice thickness and its associated sparse anatomical sampling of the patient-specific brain anatomy. This inhibits a potential approach of performing first an intra-stack slice alignment followed by a subsequent volume-to-volume registration to the reference image. Taking advantage of the valuable information on the skull geometry captured by the later reference image with similar appearance, we propose leveraging the combined information of both reference and historical slice neighbour data by deploying a slice-to-volume registration framework based on regularised motion and affine intensity correction. The regularised slice-based intensity correction is meant to deal with the intensity variations across slices and films and to balance existing intensity discrepancies to the reference image. A previous *global* intensity correction step will be vital to eliminate background noise of the historical slices and to scale the scanned image intensities accordingly. Finally, we make use of a well-established, isotropic total variation deconvolution step (Beck and Teboulle, 2009, Rudin et al., 1992) to alleviate the blurring of the historical slices resulting from both printing and scanning steps and to reduce the impact of the original point-spread-function (PSF) from the MR scanner during acquisition time.

**Fig. 2:**
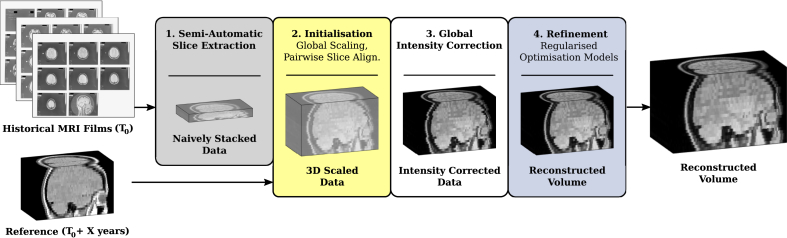
Overview of volumetric reconstruction framework for historical MR film data. Provided the scans of the MR films acquired at time *T*_0_ and a more recent, digital 3D scan of the same patient acquired *X* years later, the proposed algorithm reconstructs the volumetric representation of the original MR image acquisition at *T*_0_. Further details on the semi-automatic slice extraction, initialisation and refinement steps are visualised in Fig. 3, Fig. 4, Fig. 5, respectively.

Based on those assumptions, the proposed algorithm, illustrated in Fig. 2, reconstructs the 3D geometry of the original shape as captured by the MR films such as shown in Fig. 1.

### 2.1 Semi-automatic slice extraction

**Fig. 3:**
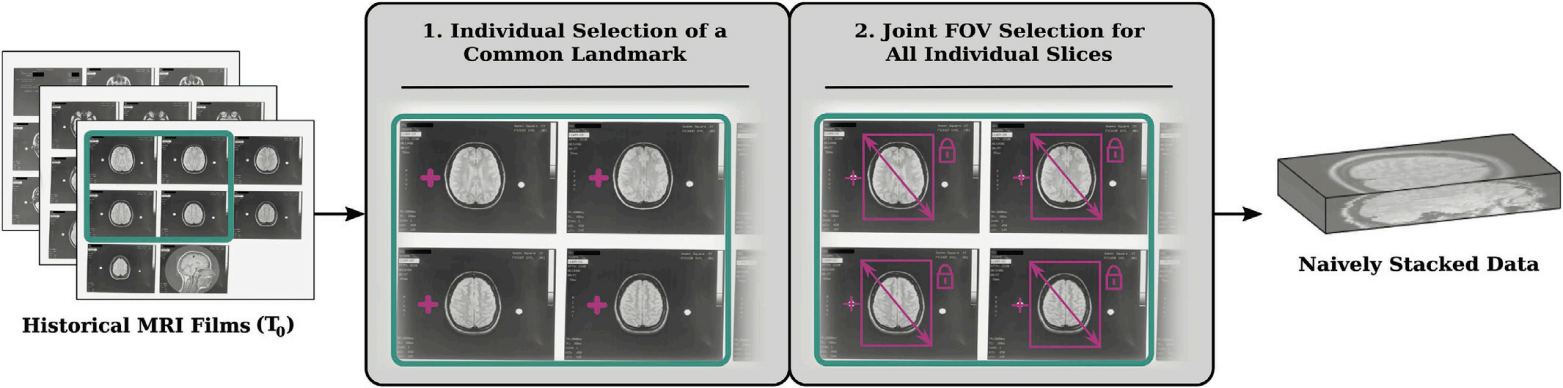
Semi-Automatic Slice Extraction. By providing the scanned MR films associated with the same acquisition a semi-automatic slice extraction step is deployed to naively stack each extracted 2D slice into a 3D stack. No meta-data information is assigned at this stage.

A flexible semi-automatic procedure is proposed to extract each individual slice-acquisition from every MR film associated with the same acquisition to address the variety of existing films and styles as pointed out in Fig. 1. As shown in Fig. 3, the first MR film is read and a common landmark is selected manually with one click per slice on the film. The selection order of the landmarks defines the slice extraction order for the later slice stacking. This ensures that the proposed slice extraction tool can deal with historical MR films where consecutive acquisitions are not necessarily printed sequentially and in the same manner across different film types. We emphasise that the manual landmark selection does not need to be very precise and is used for initialisation purposes of the motion correction algorithm only. After landmark selection, a selection window is automatically overlaid, based on default values relative to each landmark, indicating the FOV for slice extraction. The window size and respective offset are then adjusted manually, in a uniform manner, so that changes applied to one window are automatically adjusted to the rest. A more precise extraction window is then achieved, comprising the region of interest for all slices on the film. This adjustment also allows to easily extract slices even in cases where skulls are overlapping as shown in Fig. 1a. The final FOV windowing is stored and used for the subsequent films. After having marked the same common landmark on all slices on all remaining films sequentially, the selected 2D slices are extracted and stacked automatically to form a naively stacked 3D data.

### 2.2 Joint regularised motion and intensity correction model

We propose using an in-plane, affine spatial transformation model to strike a balance between fully compensating the distortions of each slice while preventing the introduction of additional image artefacts. However, due to the sparse, thick slice anatomical sampling, additional information is required to estimate the true, original, anatomical shape. The missing information can be contributed by the reference image. Due to the mentioned morphological changes of this later scan, we will use only the information around the skull; the structure which is believed to change the least over time. Hence, a good estimate of the correct slice position and geometry is likely to be found if each historical slice *y*_*k*_ matches both a corresponding reference (oblique) slice *r*_*k*_ around the skull and its immediate neighbouring slices over the FOV to achieve a good geometric consistency driven by the original MR film data. Thus, the idea is to find the slice transformation parameters $\theta_{k}\;{\in R}^{6}$ to an in-plane 2D affine spatial transformation $T\left(\theta_{k},\cdot \right):\;\Omega \;{\subset \;R}^{2}{\to R}^{2}$ which jointly minimises the costs

(1) $$\begin{array}{cc} s_{1}\left(y_{k}\left(T\left(\theta_{k},\xi \right)\right),\;r_{k}\left(\xi \right)\right)\;\forall \xi \in \Omega_{\text{skull}}\;\subset \;\Omega , & \end{array}$$

(2) $$\begin{array}{cc} s_{2}\left(y_{k}\left(T\left(\theta_{k},\xi \right)\right),\;y_{k+1}\left(T\left(\theta_{k+1},\xi \right)\right)\right)\;\forall \xi \in \Omega , & \end{array}$$

for adequate similarity measures *s*_1_, *s*_2_ for all *K* slices. To compensate intensity variations across historical slices and between historical and reference slice, a global intensity correction step, as shown in Fig. 2, and a local affine intensity compensation model during registration will be deployed. By defining the joint motion and intensity correction parameter $\Theta_{k}:=\left(\theta_{k},\;\alpha_{k},\;\beta_{k}\right)$ the cost (2), measuring the dissimilarity of neighbouring, historical slices, is defined as

(3) $$\begin{array}{ccc} N_{k}\left(\Theta_{k},\Theta_{k+1}\right):=\underset{\xi \in \Omega }{\sum }\varrho_{\gamma }\left(\alpha_{k}\;y_{k}\left(T\left(\theta_{k},\;\xi \right)\right)+\beta_{k}-\alpha_{k+1}\;y_{k+1}\left(T\left(\theta_{k+1},\xi \right)\right)-\beta_{k+1}\right) & & \end{array}$$

with a loss function $\varrho_{\gamma }:\;R\;{\to \;R}^{+},\;e\;\mapsto \;\varrho_{\gamma }\left(e\right):=\sqrt{\gamma^{2}+e^{2}}-\gamma ,$ as a smooth $\ell^{1}$-approximation with scaling factor γ>0, similar to the Huber function, to allow for a more robust optimisation. Similarly, the cost (1) between historical and corresponding reference slices is defined as

(4) $$R_{k}\left(\Theta_{k}\right):=\underset{\xi \in \Omega_{\text{skull}}}{\sum }\varrho_{\gamma }\left(\alpha_{k}\;y_{k}\left(T\left(\theta_{k},\;\xi \right)\right)+\beta_{k}-r_{k}\left(\xi \right)\right)$$

whereby only a neighbourhood around the skull $\Omega_{\text{skull}}\;\subset \;\Omega$ is considered. With $\Theta :=\left(\Theta_{1},\ldots ,\Theta_{K}\right)$ denoting the joint set of optimisation parameters and $P\left(\Theta \right):=\sum_{\theta \in \Theta }\varrho_{\gamma }\left(\theta \right)$, the motion correction problem for one stack of semi-automatically extracted slices can subsequently be written as a joint, regularised minimisation problem

(5) $$\min_{\Theta }\left(\lambda_{R}\;\overset{K}{\underset{k=1}{\sum }}R_{k}\left(\Theta_{k}\right)+\lambda_{N}\;\overset{K-1}{\underset{k=1}{\sum }}N_{k}\left(\Theta_{k},\Theta_{k+1}\right)+\lambda_{P}\;P\left(\Theta -\Theta_{0}\right)\right)$$

with weights $\lambda_{R},\;\lambda_{N}>0$, regularisation parameter $\lambda_{P}>0$ and prior $\Theta_{0}$ on the parameters which need to be defined accordingly. The critical point is to get the corresponding reference (oblique) slices $r_{k},\;k=1,\;\ldots ,\;K,$ to initialise (5).

### 2.3 Initialisation of volumetric reconstruction algorithm

**Fig. 4:**
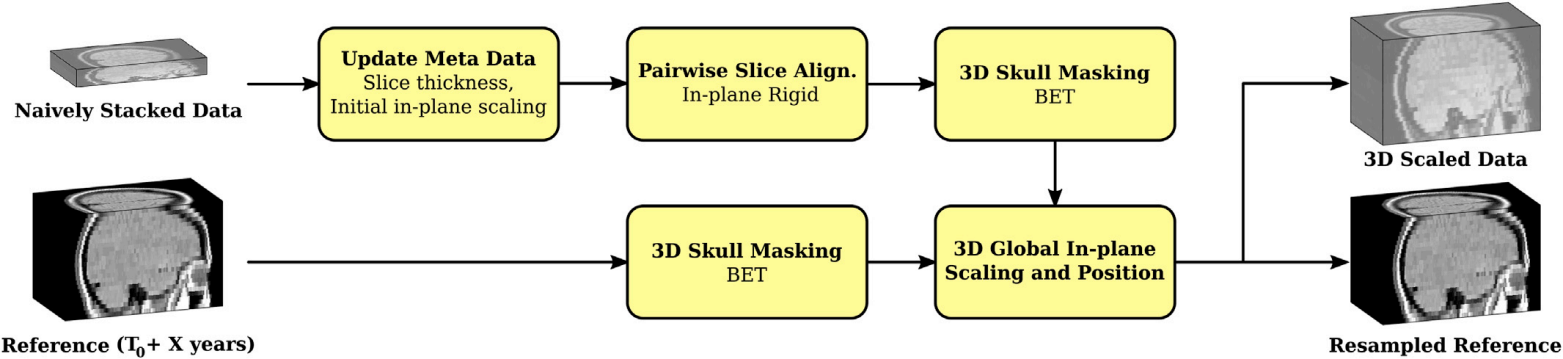
Initialisation of volumetric reconstruction algorithm: Global rescaling and positioning of the historical slices to initialise the regularised optimisation models.

As mentioned earlier, the slice thickness is the only preserved meta-data information of the slice acquisitions stated on the MR films. After the scanning, the image data is only given in pixel dimensions. Hence, the semi-automatically extracted and naively stacked slices need to be re-scaled and aligned with the reference 3D volume in order to extract the respective reference (oblique) slices *r*_*k*_. Our proposed approach is shown in Fig. 4. The slice-thickness is updated according to the information from the MR film and the in-plane scaling is initialised by a uniform value manually fixed. A subsequent rigid in-plane registration step with inter-slice regularisation is performed using the sum of the slice neighbour-terms (3) to obtain a more consistent brain geometry and to correct for possible inaccuracies of the semi-automatic slice extraction. More information on the parametrisation of the registration parameters is provided in Section 3.2. By using the Brain Extraction Tool (BET) (Smith, 2002) a mask surrounding the skull of both the in-plane registered stack and the reference image is created. A subsequent in-plane 3D similarity registration based on normalised cross-correlation aligns the entire stack with the reference and estimates a uniform in-plane scaling factor for all slices to match their skull masks. The additionally resampled reference image to the 3D scaled stack space both provides the oblique slices *r*_*k*_ and serves for the subsequent global intensity correction step.

### 2.4 Global intensity correction

Due to the printing, storage over years and scanning, the historical slices may carry a substantial amount of background noise and have low image contrast. A global intensity correction step aims to improve the intensity contrast by using the resampled 3D reference image intensity information and to keep the slice intensities as close as possible to the original ones’ at the same time. With $q_{20\text{\%}}$ being the global 20%-intensity percentile of all historical slices, all slice intensities i are capped via $i\leftarrow \max\left(i-q_{20\text{\%}},0\right)$ to eliminate background noise whereby the 20%-threshold was found experimentally by visual analysis. We then apply a uniform-across-slices linear intensity correction step.

### 2.5 Refinement of volumetric reconstruction

**Fig. 5:**
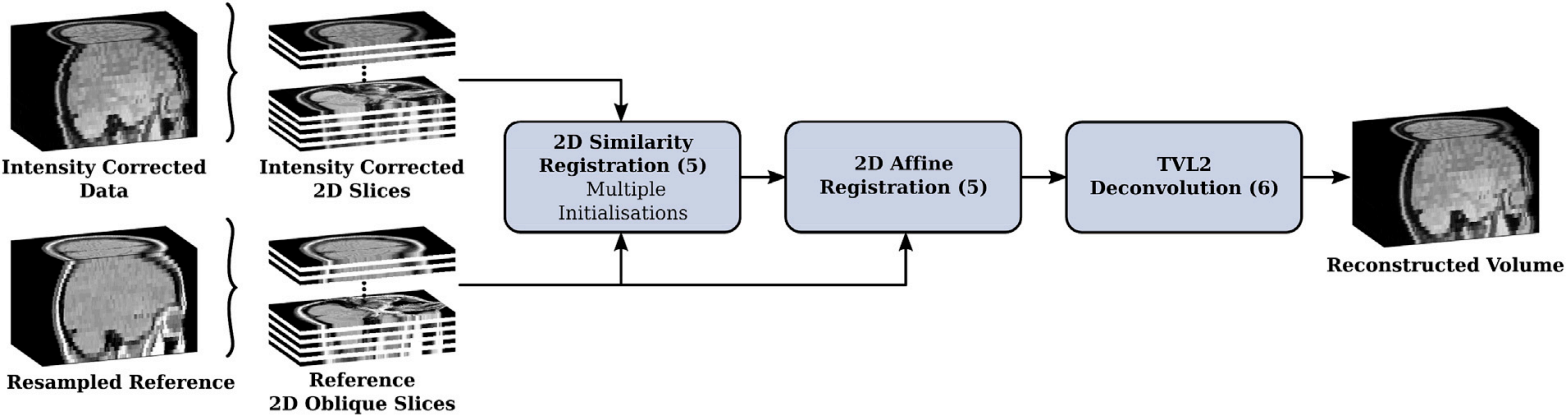
Refinement of volumetric reconstruction algorithm: Application of joint, regularised motion and intensity correction (5) in addition to the TVL2-deconvolution (6).

In order to increase the convergence basin of the joint-regularised registration model (5), a step-wise increase in transformation complexity is chosen for the **slice-based motion correction** step to correct for individual geometric distortions, illustrated in Fig. 5. A similarity 2D transformation is used first in (5) to correct for rigid motion and uniform in-plane scaling for each slice separately. This is performed twice with different spatial initialisation transformations, i.e. using the identity transformation and the initialisation transformation based on the moments of the skull-masked historical and the reference slice whereby the result with overall lower cost is selected. The prior term in (5) is chosen to penalise in-plane scaling and intensity coefficients. Since all slices have been uniformly scaled previously, the prior value for in-plane scaling is set to 1. Similarly, due to the global intensity scaling, the coefficients *α*_*k*_ and *β*_*k*_ are expected to be close to 1 and 0, respectively. Regularisation parameters are found experimentally and described in Section 3.2. Afterwards, the full, 2D affine transformation model is chosen for (5) so that the historical slice can match the skull as accurately as possible.

After having estimated the positions and geometrical properties of the slices a subsequent image deconvolution step is performed for each slice separately in order to restore each individual slice without mixing neighbouring slice information. For this purpose, we rely on a 2D **isotropic total variation (TVL2) deconvolution** step (Beck and Teboulle, 2009, Rudin et al., 1992)

(6) $$\underset{\text{x}}{\min}\left(\frac{1}{2}||y-A\left(\sigma^{2}\right)x|{|}_{\ell^{2}}^{2}+{\lambda \;\mathrm{TV}}_{\text{iso}}\left(x\right)\right)\;\text{s}\text{.t}\text{.}\;x\geq 0$$

for each individual slice $y\in \left\{y_{1},\;\ldots ,y_{K}\right\}$ with $y_{k}={\left(y_{k}\left(\xi \right)\right)}_{\xi \in \Omega }$ representing the vectorised slice, x its deblurred version, **A** the blurring operator with covariance *σ*^2^ to jointly describe the in-plane blurring of the image acquisition, printing and scanning and λ>0 the regularisation parameter. A *matrix-free* implementation is chosen in order to avoid the storage of large matrices (Diamond and Boyd, 2015). The optimisation problem (6) is solved via Alternating Direction Method of Multipliers (ADMM) described in Boyd et al. (2011). The implemented, scaled, explicit form of ADMM iterations reads

(7) $$x^{i+1}:=\underset{x}{\arg\;\min}\left(\frac{1}{2}||y-Ax|{|}_{\ell^{2}}^{2}+\frac{\rho }{2}||\nabla x-v^{i}+w^{i}|{|}_{\ell^{2}}^{2}\right)\;\text{s}\text{.t}\text{.}\;x\geq 0$$

(8) $$v^{i+1}:=S_{\lambda ∕\rho }\left(\nabla x^{i+1}+w^{i}\right)$$

(9) $$w^{i+1}:=w^{i}+\left(\nabla x^{i+1}-v^{i+1}\right)$$

with the auxiliary variable v, the scaled dual variable w, the Lagrange multiplier *ρ* and the vectorial soft threshold operator $S_{\lambda ∕\rho }$ (Boyd et al., 2011). Given that the standard Lawson & Hanson algorithm (Lawson and Hanson, 1974) cannot cope with large-scale non-negative least-squares problems several specialised methods have been proposed in the literature to solve minimisation problems like (7) such as presented in Becker and Fadili, 2012, Kim et al., 2010a, Kim et al., 2013. In this work, we used the L-BFGS-B v3.0 solver (Byrd et al., 1995, Morales and Nocedal, 2011) which, although not specifically designed for non-negative least squares, generally shows good performance for such problems and, especially for large-scale problems, regularly outperforms other modern methods (Kim et al., 2010a).

## 3 Data, evaluation methodology and results

### 3.1 Data

A cohort of people recruited soon after a CIS was first assessed at the National Hospital for Neurology and Neurosurgery, Queen Square, London, between 1984 and 1987 and followed up at regular time points until present (Miller et al., 1988, Miller et al., 1989, Morrissey et al., 1993, O'Riordan et al., 1998, Brex et al., 2002, Fisniku et al., 2008); a 30-year longitudinal follow-up, clinical study is currently underway including more than 100 image acquisitions captured on historical films. The preserved MR films were scanned using the Vidar DiagnosticPRO Advantage film digitizer, processed with the Hipax Diagnostic Workstation medical image viewer software and exported to DICOM files. For the current study, a subset of 20 MR film sets (18 acquisitions at 5-year follow-up acquired at 0.5T and two at 10-year follow-up acquired at 1.5T) was available where both the original MR films and the stacked, digital scans of the exact same 5 mm thick PD-like slice acquisitions were available. This is a rare situation and allows to validate the volumetric reconstruction pipeline against ground-truth data. In 18 out of those 20 subjects, the acquisitions were captured on films where two consecutive slices showed overlapping skull and brain structures similar to Fig. 1a and d. To recover the spatial correspondences for each slice a later PD-scan of the same subject was used as the reference which is typically available in longitudinal studies. The reference scan was acquired as stacks of 5 mm thick slices and, depending on the subject-specific follow-ups and availability, either was a 10-, 14- or 20-year time point after the baseline scan of the same subject. This reference was also used to correct for existing left-right flipping of the brain we encountered in the scans.

### 3.2 Parametrisation of volumetric reconstruction pipeline

The entire, regularised volumetric reconstruction framework was implemented in Python while taking advantage of ITK for the individual registration steps. The joint regularised registration problem (5) was implemented via the least_squares algorithm of SciPy where the exact Jacobian was provided for both accelerated and more accurate computational results. The framework is made open source and available on github.^2^

The semi-automatic slice extraction tool stores the naively stacked 3D data as a NIfTI image for further processing. The rigid in-plane registration step with inter-slice regularisation using slice neighbour-terms (3) was initialised based on the slice moments and used least-squares differences as similarity metric whereby 10 iterations were performed in the least_squares algorithm. By considering this stack of neighbour-aligned slices as a 3D volume BET was applied to extract its brain mask. The skull mask was then defined as its negated mask followed by a subsequent dilation step to account for geometric discontinuities across slices. The skull mask for the more recent 3D reference image was obtained analogously but without the dilation step. Constrained by the skull masks, the in-plane 3D similarity registration step was performed using cross-correlation as the similarity measure, linear interpolation resampling, regular step gradient descent optimiser with physical shift scales estimation and a three level multi-resolution framework which was initialised by a previously performed rigid registration based on the respective 3D image moments.

The global intensity correction was performed as described in Section 2.4 by using the global 20%-intensity percentile for the lower threshold. This threshold was found experimentally by visual comparisons.

The motion correction method with inter-slice regularisation and reference image information transfer in combination with the affine intensity correction model (5) described in Section 2.5 was implemented via the least_squares optimiser. The prior term P was set up to regularize the in-plane scaling and the affine intensity correction parameters only so as to extend the inter-slice regularisation of the motion correction framework. Due to the global scaling and intensity correction performed during the initialisation steps, the associated prior values are set to 1 for the in-plane scaling and *α_k_*_0_ = 0 and *β_k_*_0_ = 1 for the intensity correction parameters, respectively. By using the solver-specific soft_l1 for the respective λ-weighted residuals in (5), the applied smooth $\ell^{1}$-approximation corresponds to $\varrho_{1∕\lambda }\left(e\right)=\sqrt{1∕\lambda^{2}+e^{2}}-1∕\lambda$ for $\lambda \in \left\{\lambda_{R},\;\lambda_{N},\;\lambda_{P}\right\}$. The weights and the regularisation parameter were found experimentally and set to $\lambda_{N}=1,\;\lambda_{R}=10$ and $\lambda_{P}=10^{6}$ in (5) for the 2D similarity registration step, respectively, whereby 10 iterations were performed. For the subsequent 2D affine registration, the regularisation parameter was reduced to $\lambda_{P}=10^{3}$ and 20 iterations were performed which was sufficient to achieve overall convergence. During experiments we found that omitting the inter-slice regularisation term in (5) can lead to severe misregistrations during motion correction. We, therefore, conclude that the proposed motion correction framework based on inter-slice regularisation is key in order to reliably achieve volumetric reconstructions of high anatomical accuracy. Associated comparisons are provided in the supplementary material. For the TV reconstruction step (6), the regularisation parameter *λ* = 5, the Lagrange multiplier *ρ* = 0.5 and the covariance *σ*^2^ = 0.25 for the blurring operator A were found via L-curve studies. The first-order Tikhonov problem (7) in the corresponding TVL2 deconvolution step with its positivity constraints was solved via the L-BFGS-B algorithm of SciPy to iteratively solve for the unique minimizer whereby 10 ADMM iterations were performed.

### 3.3 Evaluation methodology

**Fig. 6:**
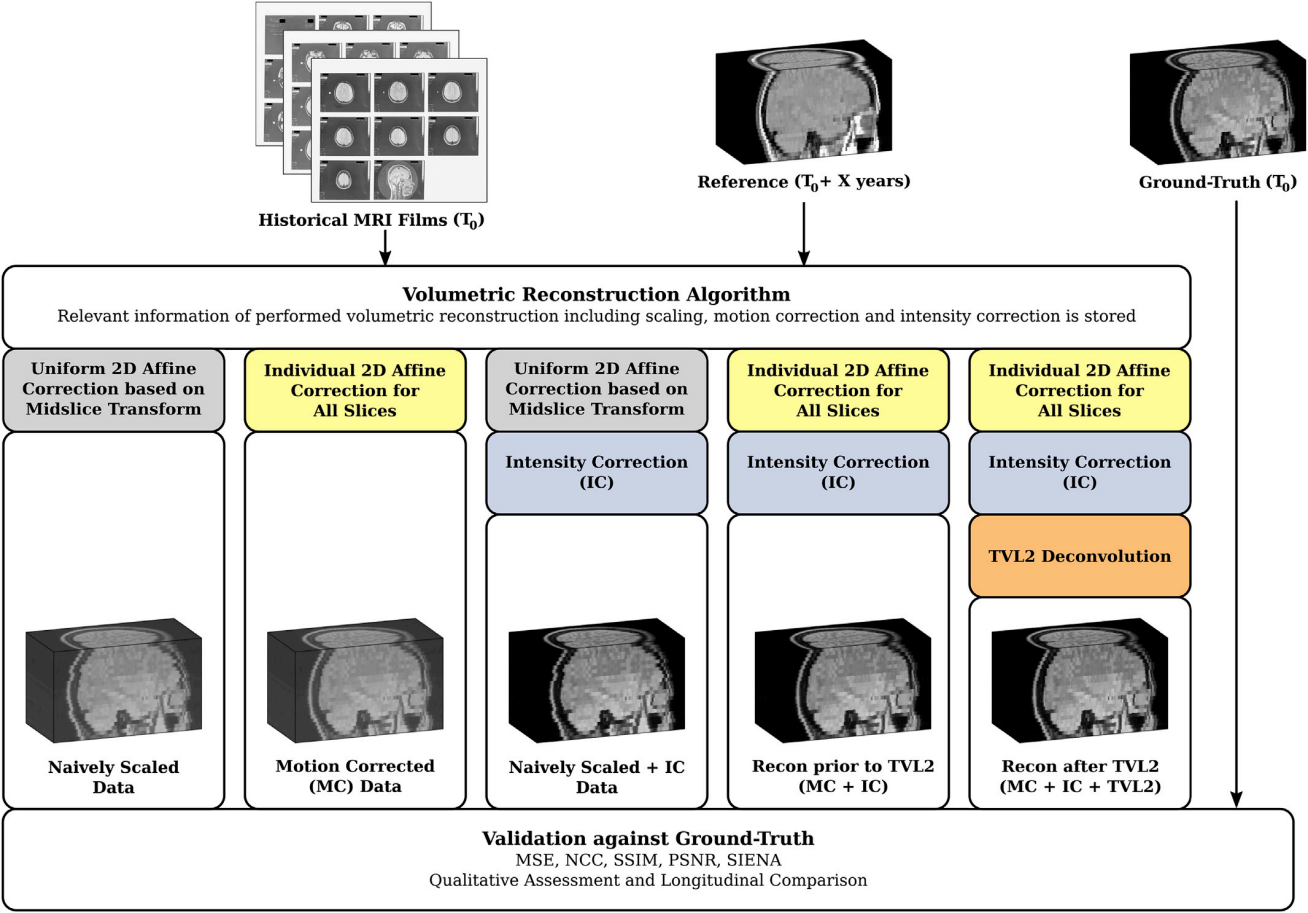
Summary of the pipeline used to validate the volumetric reconstruction framework. Each image shows a typical volumetric output obtained after the respective steps of processing.

Several similarity measures like mean squared error (MSE), peak signal-to-noise ratio (PSNR), structural similarity index measure (SSIM) (Wang et al., 2004) and normalised cross-correlation (NCC), were used to assess the similarity between ground-truth and different intermediate results until the final volumetric reconstruction with the TVL2 step. We considered the reconstruction prior to the TVL2 step (full 2D affine correction including intensity correction but no TVL2 step), the motion corrected (MC) data (full 2D affine correction but no intensity correction), the naively scaled data (naively stacked data scaled based on the final 2D affine transformation belonging to the mid-slice of the stack but no intensity correction), the naively scaled and intensity corrected (IC) data (same intensity correction applied to the naively scaled data), and the reference used for motion and intensity correction. A visual summary of the used validation pipeline including the respective short-hands is shown in Fig. 6. The similarity measures were only evaluated at the masked brain region obtained via BET (Smith, 2002) applied on the ground-truth. The required alignment of stacks prior to the evaluation was obtained by using the rigid registration algorithm reg_aladin within NiftyReg^3^ which is based on block-matching (Modat et al., 2014).

In addition, we evaluated the absolute mean surface motion in linear voxel units of the reconstruction after TVL2 step and the naively scaled and intensity corrected data compared to the ground-truth which reflects the sum of all edge motions between two segmentations (Smith et al., 2002). This measure was computed via Structural Image Evaluation, using Normalization, of Atrophy (SIENA) (Smith et al., 2002), where we only measured in-plane edge motion because of the missing top brain on the historical data, as visible in Fig. 2.

Following this, a subjective quality assessment in a clinical context was performed where two blinded neurologists assessed the naively scaled and intensity corrected data, the reconstruction prior to TVL2 and the reconstruction after TVL2 step side-by-side and in comparison with the naively scaled data, the ground-truth data and the reference image used for motion and intensity correction. After performing a contrast auto-adjustment in the image viewer for more comparable visualisation, scores were given for:

1. Clinical usefulness ranking based on lesions' conspicuity and geometric plausibility/skull continuity essential for volumetric measurements in addition to a final score on overall preference.
2. Ground-truth comparison in terms of interpretability based on image quality and anatomical similarity.

### 3.4 Results

**Fig. 7:**
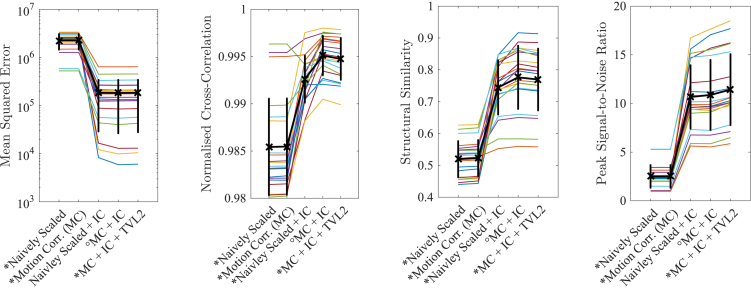
Similarity measures evaluated at the ground-truth brain for each subject separately. The black curve shows the error bar given by mean and standard deviation. A ^∗^ indicates that the reconstructions are statistically significantly different from the reconstruction prior to TVL2 (MC + IC, marked with $a^{\circ }$) based on a paired *t*-test (p<0.05) and Bonferroni-correction.

Fig. 7 illustrates that the biggest improvement in measured similarity is achieved by the intensity correction step. Importantly, however, motion correction is shown to significantly increase the similarity to the ground-truth. A further, significant improvement in PSNR is achieved by the TVL2 deconvolution step at the expense of measured similarity with the ground-truth. This can be explained by the fact that the considered ground-truth stack is affected by blurring and noise due to the acquisition performed decades ago. Therefore, deblurring can counteract the PSF during acquisition time and have a positive impact on the image quality (Beck and Teboulle, 2009, Rudin et al., 1992, Buades et al., 2005). The evaluation in Table 1 allows a more detailed assessment of the contribution of each individual step as outlined in Fig. 6. It clearly shows that motion correction applied on both intensity and non-intensity corrected data leads to significant improvements in measured image similarities. It also illustrates the high intensity contrast dependency of all involved measures which explains the visually striking impact of the performed non-linear intensity correction observed in Fig. 7. However, by considering the same starting point of either non-intensity or intensity corrected data, the significant similarity improvements by the performed motion correction underline its importance and effectiveness to obtain high-quality volumetric reconstructions.

**Table 1:** Summary of similarity measures evaluated at the ground-truth brain stated as mean and standard deviation for all 20 subjects. The MSE was omitted in favour of less absolute intensity value sensitive measures. The symbol $\neq^{*}$ indicates a statistically significant difference between the left and right hand-side with respect to the statistics shown in the sub-index based on a paired *t*-test (p<0.05) and Bonferroni correction.

	NCC	SSIM	PSNR	Notes
a) Naively Scaled	0.985 ± 0.005	0.519 ± 0.056	2.505 ± 1.159	
b) Motion Corr. (MC)	0.985 ± 0.005	0.523 ± 0.056	2.503 ± 1.160	b $\neq_{\text{NCC/SSIM}}^{*}$ a
c) Naively Scaled + IC	0.993 ± 0.002	0.745 ± 0.085	10.671 ± 3.250	c $\neq_{\text{NCC/SSIM/PSNR}}^{*}$ a,b
d) MC + IC	0.995 ± 0.002	0.776 ± 0.099	10.876 ± 3.589	d $\neq_{\text{NCC/SSIM}}^{*}$ a–c & d $\neq_{\text{PSNR}}^{*}$ a,b
e) MC + IC + TVL2	0.995 ± 0.002	0.770 ± 0.097	11.426 ± 3.639	e $\neq_{\text{NCC/SSIM/PSNR}}^{*}$ a–d

**Fig. 8:**
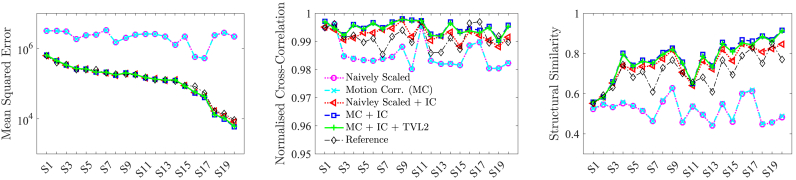
Similarity measures evaluated at the ground-truth brain for each individual subject. The subjects on the horizontal axis are ranked according to the MSE of the reference image, shown for comparison purposes.

Fig. 8 provides a per-subject comparison and shows the impact of each performed step in the volumetric reconstruction pipeline for each individual case. The high figures in NCC and SSIM of the naively scaled and intensity corrected data reveal that the semi-automatic slice-extraction is able to achieve an overall high accuracy of initial slice alignment which is further, significantly, improved by the volumetric reconstruction pipeline. The MSE suggests that the performed intensity correction is very effective for all subjects and yields slice intensities similar to their respective references. The figures of NCC and SSIM confirm that the motion correction had a significant impact and led to substantial improvements in image similarity for almost all subjects. Both NCC and SSIM also show that our volumetric reconstructions achieve higher similarity than the reference images illustrating the morphological changes the brain has undergone over time and differences owed to the different contrast.

SIENA measured the absolute mean surface motion between ground-truth and naively scaled image as 0.80±0.20 linear voxel units with 95%-confidence interval (CI) from 0.71 to 0.88. The reconstruction after TVL2 step achieved a mean figure of 0.61±0.13 with 95%-CI from 0.56 to 0.67 which corresponds to a significant improvement based on paired *t*-test (p<0.05) of about 25%. Therefore, detected edge-motion was significantly reduced which underlines the capability of the performed motion correction framework to significantly increase the accuracy of the obtained volumetric reconstructions.

To further investigate the impact of the performed motion correction and denoising steps we analysed the reconstruction quality of the associated volumes and in direct comparison with the naively scaled and intensity corrected data by performing a qualitative assessment by expert neurologists. The neurologist's evaluation, shown in Table 2, indicates that the blinded neurologists had a clear preference for our volumetric reconstructions over the naively scaled data given their higher geometric plausibility and improved lesions' conspicuity. Adding the TVL2 deconvolution step yields even further improvement. Particularly, the score on geometric plausibility states that the performed motion correction always yielded an improved outcome. In direct comparison, we almost always achieve results which are visually indistinguishable from the ground-truth data. In addition, it was felt that especially the reconstruction after TVL2 step gives rise to improved interpretation; better than the original, non-processed ground-truth data which itself is affected by its PSF-affected physical acquisition from the past.

**Table 2:** Summary of blinded, clinical evaluation averaged over all 20 subjects. Lesions conspicuity and geometric plausibility/skull continuity subjectively rank preferred reconstruction from 1 (least preferred) to 3 (most preferred). Preferred image score indicates the number of times the respective reconstruction was the preferred choice. Ties were allowed for the geometric plausibility and preference ranking in case images were visually indistinguishable. The anatomical similarity to the ground-truth is rated 0 (distinguishable) or 1 (not distinguishable). Image quality similarity to ground-truth scores are 0 (worse, but interpretable), 1 (same as ground-truth) and 2 (improved interpretation).

	Clinical Usefulness Ranking			Ground-Truth Comparison	
	Lesions' Conspicuity	Geometric Plausibility	Pref. Image	Anatomical Similarity	Image Quality
Naively Scaled + IC	1.45 ± 0.69	2.00 ± 0.00	0	0.10 ± 0.31	0.70 ± 0.73
MC + IC	2.40 ± 0.50	3.00 ± 0.00	8	0.80 ± 0.41	1.35 ± 0.59
MC + IC + TVL2	2.85 ± 0.37	3.00 ± 0.00	15	0.95 ± 0.22	1.50 ± 0.51

**Fig. 9:**
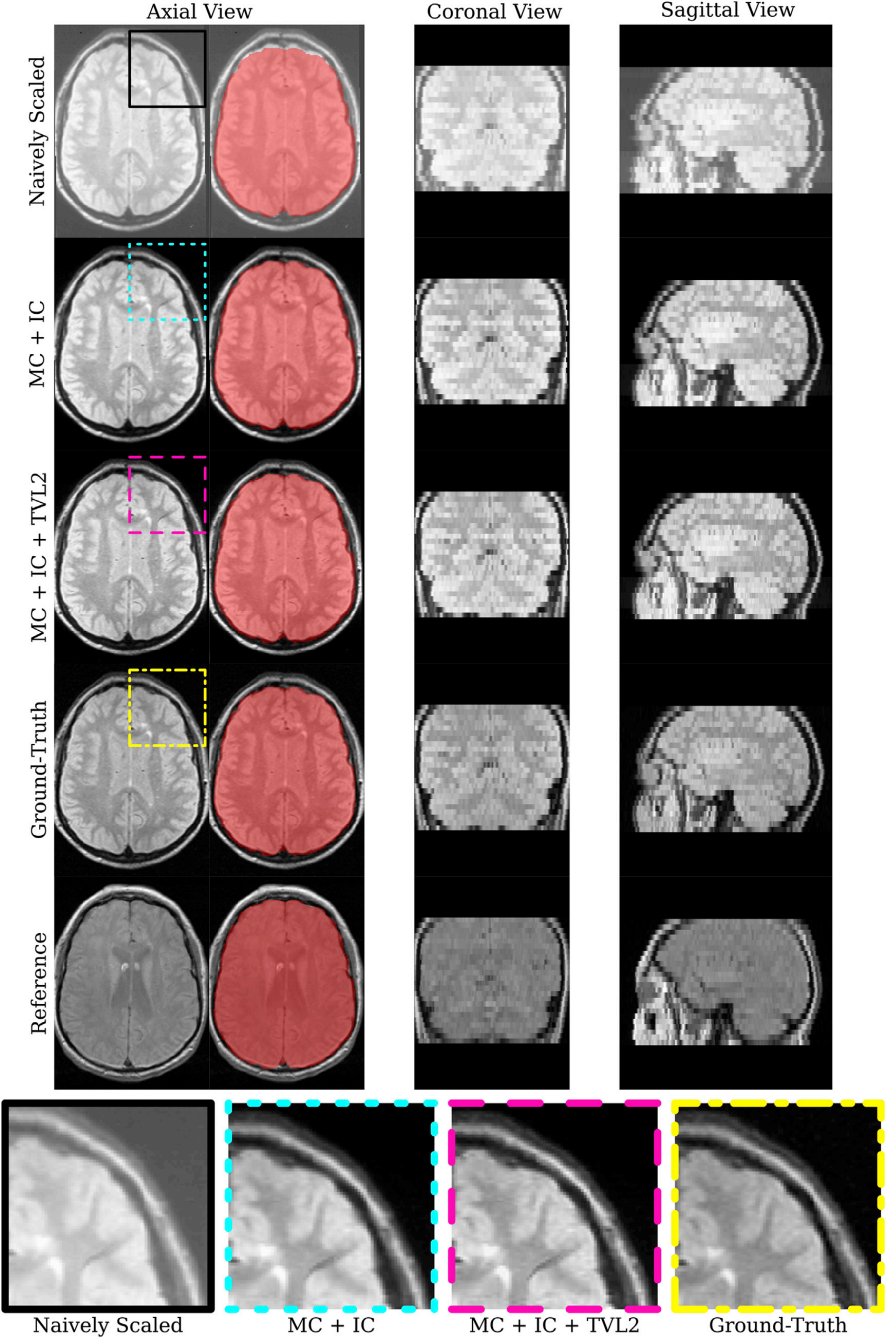
Visual comparison between reconstruction results and original data. The automatically segmented ground-truth brain is shown as a red overlay in each of the images and illustrates the reconstruction accuracy of the obtained volumetric reconstruction from limited FOV data. At the bottom, a zoomed window highlights the improvement and reconstruction quality. There, also a stroke of dirt is visible on the historical data.

In Fig. 9 the naively scaled stack and the reconstruction results before and after the TVL2 step are provided for one of the cases along with the ground-truth data and the reference image used for motion and intensity correction. This example was selected to showcase the result for one of the visually most motion corrupted stacks after the semi-automatic slice extraction step which served as initialisation of the volumetric reconstruction framework. The overlaid brain mask of the ground-truth illustrates the discrepancy of the naively scaled data which becomes almost invisible in the obtained volumetric reconstruction results. Only at the neck, slight inaccuracies of the reconstructions become apparent in the sagittal view which can be explained by the high intensities at this region on the reference image. The bottom row of Fig. 9 shows a zoomed-in comparison highlighting the high accuracy of the motion correction in combination with the image quality improvements due to intensity correction and deconvolution steps.

**Fig. 10:**
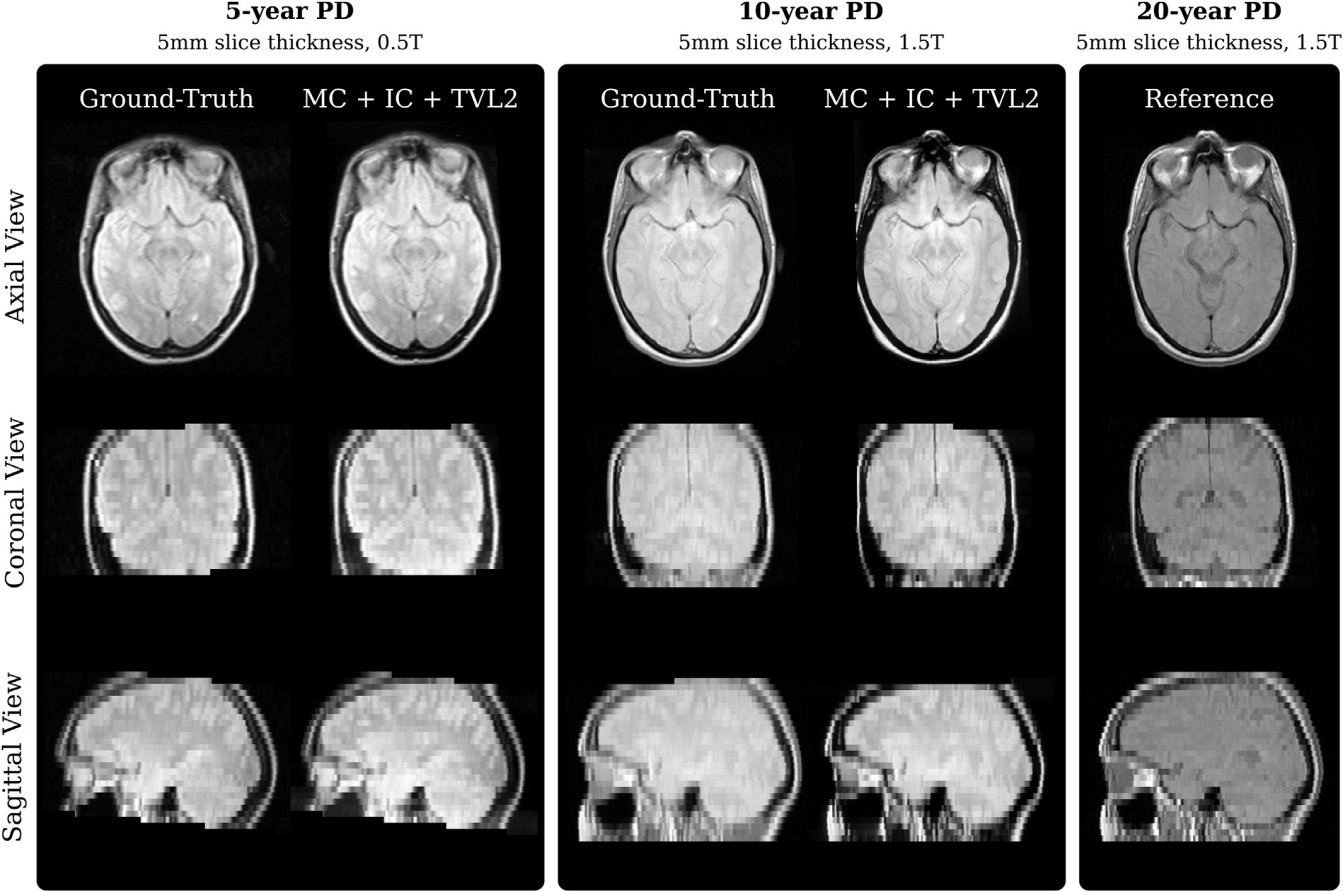
Qualitative comparison of three longitudinal scans used in this study associated with a subject where the baseline scan was acquired in 1986. The comparison shows the 20-year scan used as reference image for the volumetric reconstruction pipeline along with the linearly resampled digital, ground-truth data, and the obtained volumetric reconstructions from the historical films of the respective 5-year and 10-year follow-up time points. Visually, differences between the reconstructions and the ground-truth are hardly detectable. The measured ground-truth-similarities for the reconstructed 5-year scan are 0.992, 0.787, 10.294, for NCC, SSIM and PSNR, respectively. Similarly, the respective figures for the 10-year scan are 0.993, 0.884 and 16.167. Note that due to overlapping skulls on the historical MR films, see Fig. 1a, only the part visible on the films could be recovered during the semi-automatic slice extraction step for the 10-year scan.

Fig. 10 provides a qualitative comparison of three longitudinal scans used in this study associated with a subject where the baseline scan was acquired in 1986. The visualised subject represents the only available case in this study where both a 5-year and 10-year digital stack, i.e. a ”ground-truth”, were available. One can observe the highly consistent reconstructions obtained by the proposed volumetric reconstruction framework with closely matching contours of skull and brain for both 5- and 10-year follow-up scans.

Using our non-optimised implementation, the typical processing time to restore one single subject from printed MR films was measured to be approximately 1h 20min on a single computer. This includes about 2 min to 5 min of user interaction to operate the semi-automatic slice extraction tool. The remaining processes are fully automated whereby the volumetric reconstruction steps, including motion and intensity correction, were measured to take about 45min and the final TVL2 deconvolution step about 30min.

## 4 Discussion

In this work, we present, and to the best of our knowledge for the first time, a framework which reconstructs the volumetric stack from printed, historical, limited FOV MR films being acquired decades earlier. The proposed semi-automatic slice-extraction algorithm is capable of dealing with different MR films of many kinds, formats and appearances including films where parts of the brain and skull are overlapping on consecutively printed slices. Its particular design choice ensures the robustness to any acquisition set-up with respect to slice-ordering or single/multi-slice acquisition in the sense that printed slices which capture adjacent anatomy are reliably combined to form a first naively stacked 3D data for further processing regardless the encountered type of historical MR film. We introduce a joint slice-to-volume registration with inter-slice transformation regularisation and slice intensity correction based on a smooth $\ell^{1}$-approximation as loss functional for a more robust registration framework. We put a particular focus on using methods which are able to restore the original image quality and geometry of the historical scans without introducing additional image artefacts. We emphasise on numerical accuracy and computational efficiency by providing the exact Jacobian for all cost functions and use a matrix-free implementation during the TVL2 deconvolution step. We test our reconstruction results against ground-truth data both quantitatively and in a clinical context and demonstrate the high reconstruction quality and suitability of the proposed framework.

The performed validation proves the used global intensity correction step sufficient to deal with the existing intensity differences and illustrates its substantial contribution to an overall improved similarity to the ground-truth data. It demonstrates that the motion correction algorithm yields a further, statistically significant, improvement towards overall similarity in both the measures of structural similarity and normalised cross-correlation. It underlines that the proposed motion correction is vital to eliminate implausible discontinuities across slices existing after the semi-automatic slice extraction and yields reconstructions with highly plausible brain geometries which accurately reflect the patient-specific anatomy. It illustrates that the volumetric reconstruction framework and its design is capable of robustly reconstructing accurate volumetric reconstructions from historic MR films even when skulls are merged and, consequently, information on the skull is compromised. It shows that the additional TVL2 deconvolution step gives rise to volumetric reconstructions which are visually almost indistinguishable from the ground-truth data and may even lead to an improved interpretation over the original, digital volumes.

The volumetric reconstruction algorithm with its joint, in-plane 2D affine motion and intensity correction model in addition to the in-plane 2D deconvolution step is designed as a framework to carefully balance the desire of fully recovering the original 3D image anatomy without mixing slice neighbour information or introducing image artefacts. However, this approach can account for axial motion only and may well be insufficient in cases where subject motion occurred during acquisition time or more complex motion is present in the historical MR films. The consistently high reconstruction quality shown in the course of the validation supports the argument that inter-slice subject motion was not an issue for the data in our study. Our proposed pipeline is not designed to reconstruct parts of the brain which are not visible in the original MR films. This includes anatomical information hidden by partial voluming effects due to the thick slice acquisitions or parts of the brain which are entirely missing due to a reduced FOV. Hence, a truncation at the vertex will prevent whole brain volumes from being estimated. Furthermore, the anatomical accuracy of the volumetric reconstruction depends on the estimate of the respective, oblique reference slices obtained by resampling of the reference stack. Thus, a higher resolution of the reference image would provide more scope of accurate registrations. Minor issues we encountered with our method were associated with subjects where either the top or the bottom slices of the historical stack did not have a sufficient overlap with the FOV of the reference image. For such slices only the slice neighbour-term (3) contributes to the cost function (5). This situation effectively simplifies to an intra-stack in-plane registration problem and tends to align bordering slices so that they fit their proximal neighbour. Consequently, minor inaccuracies for these slices are visible which may slightly differ from the correct anatomical shape.

This volumetric reconstruction pipeline was developed based on the assumption that a reference 3D digital image exists with similar intensity appearance in order to extract both its meta-data and intensity contrast information. However, in cases where no such reference is available the proposed volumetric reconstruction pipeline could still be applied in various ways. In case a naive digital 3D representation is sufficient for the problem at hand the naively stacked data after semi-automatic slice extraction can be used whereby the imaging meta-data could be updated with manual values. Additional motion correction could be performed by using intra-stack regularisation (3) only. However, this is likely to lead to non-physiological slice-alignments like a straight skull delineation. Alternatively, an atlas could be used to apply the entire volumetric reconstruction method as outlined in Section 2. Nevertheless, a high reconstruction accuracy depends on the possibility to realign all slices so that they match the patient-specific anatomy as closely as possible. For this step, a reference image which accurately reflects the subject anatomy is key and the higher the slice thickness becomes the more important such a similarity will be for our proposed framework.

Overall, our framework has shown its capability to accurately reconstruct 3D volumes from printed MR films of MS subjects and will help in the robust analysis of a uniquely long-term study spanning 30 years of MRI scans of people followed up after a CIS. This study includes more than 100 subject scans captured on historical films which can be reconstructed with our proposed technique for further image processing and analysis. However, applications of the proposed method are not confined to CIS and MS studies, and it may prove useful for the longitudinal assessment of lesions and anatomical structures in a variety of other conditions that affect the brain. Our framework may also be useful in current clinical practice, where not uncommonly, patients have had MR imaging performed previously on different scanners, and the only format available is in film format. The volumetric reconstruction of these images would allow the digital storage of the data, and also a longitudinal comparison with an up-to-date scan. Moreover, despite being tested on only PD-like images where ground-truth data was available, the proposed framework may be used for other MR image contrasts as well.

## Footnotes

1 https://github.com/gift-surg/VolumetricReconstructionFromPrintedFilms.

2 https://github.com/gift-surg/VolumetricReconstructionFromPrintedFilms.

3 https://sourceforge.net/projects/niftyreg.
