# Supplementary material for: Volumetric reconstruction from printed films: Enabling 30 year longitudinal analysis in MR neuroimaging
Source: Neuroimage. 2018 Jan 15;165:238–50. doi: 10.1016/j.neuroimage.2017.09.056 (PMC5737406; doi:10.1016/j.neuroimage.2017.09.056)
Supplement: Online data [file mmc1.pdf]

Supplementary Material

Volumetric Reconstruction from Printed Films: Enabling 30 Year Longitudinal Analysis in MR Neuroimaging

Michael Ebner, Karen K. Chung, Ferran Prados, M. Jorge Cardoso, Declan T. Chard, Tom Vercauteren, Sébastien Ourselin

In this supplementary material, we summarize additional experiments to underline the importance of using inter-slice regularisation in the proposed motion correction framework.

For this purpose, we volumetrically reconstructed the historical data described in Section 3.1 with the method described in Section 2 by using three variants of the cost function (5). Using the parametrisation of the volumetric reconstruction pipeline as described in Section 3.2, we reconstructed the historical data in three experiments characterized by increasing model complexity by varying the parameters $\lambda_{\mathcal{R}}, \lambda_{\mathcal{N}}$ and $\lambda_{\mathcal{P}}$. Specifically, we ran the reconstruction pipeline a) without using regularisation for motion correction, i.e. motion correction is based on the reference similarity term $\mathcal{R}_k$ alone, b) without using inter-slice regularisation, i.e. using the reference similarity term $\mathcal{R}_k$ and the regularisation term $\mathcal{P}$ but no inter-slice similarity term $\mathcal{N}_k$, and c) using our proposed method. The three experiments were run based on the parameters

a) $\lambda_{\mathcal{R}} = 10, \lambda_{\mathcal{N}} = 0, \lambda_{\mathcal{P}} = 0$, i.e. no regularisation at all

b) $\lambda_{\mathcal{R}} = 10, \lambda_{\mathcal{N}} = 0, \lambda_{\mathcal{P}} = 10^3$, i.e. no inter-slice regularisation

c) $\lambda_{\mathcal{R}} = 10, \lambda_{\mathcal{N}} = 1, \lambda_{\mathcal{P}} = 10^3$, i.e. method as proposed

By using a similar comparison as in Table 1, we quantitatively compared the reconstruction quality achieved by all three variants for all 20 subjects to investigate the importance of using inter-slice transform regularisation in (5). The results are summarized in Table S1 and show that the measured ground-truth similarity is consistently higher in terms of both NCC and SSIM by using the proposed method with inter-slice regularisation and outperforms any of the "reduced" methods a) and b). This can be explained by the fact that the insufficient transform regularisation of the motion correction based on a) or b) can lead to severe misregistrations of individual slices and, subsequently, to volumetric reconstructions of poor anatomical plausibility.

Additionally, we provide a qualitative comparison for one subject to illustrate the insufficiency of the methods a) and b) to reliably achieve high-quality reconstructions in Fig. S1 which summarizes our experience: Using motion correction based on inter-slice regularisation reduces the risk of slice misregistration and generally achieves volumetric reconstructions of high anatomical accuracy.

Table S1: Summary of similarity measures evaluated at the ground-truth brain stated as mean and standard deviation for all 20 subjects. The MSE was omitted in favour of less absolute intensity value sensitive measures. Bold values correspond to the best outcome. A * indicates that the reconstructions are statistically significantly different from the result obtained by the proposed method based on a paired t-test ($p < 0.05$) and Bonferroni-correction.

	**NCC**	**SSIM**	**PSNR**
**a) Motion Corr. (MC) without reg.**	0.985 ± 0.005	$0.521 \pm 0.056^*$	$\mathbf{2.505 \pm 1.155}$
**b) MC without inter-slice reg.**	0.985 ± 0.005	0.522 ± 0.056	2.503 ± 1.159
**c) MC with proposed method**	$\mathbf{0.985 \pm 0.005}$	$\mathbf{0.523 \pm 0.056}$	2.503 ± 1.160
**a) MC + IC without reg.**	0.992 ± 0.006	$0.754 \pm 0.100^*$	10.780 ± 3.285
**b) MC + IC without inter-slice reg.**	$0.994 \pm 0.002^*$	$0.758 \pm 0.093^*$	10.628 ± 3.245
**c) MC + IC with proposed method**	$\mathbf{0.995 \pm 0.002}$	$\mathbf{0.776 \pm 0.099}$	$\mathbf{10.876 \pm 3.589}$

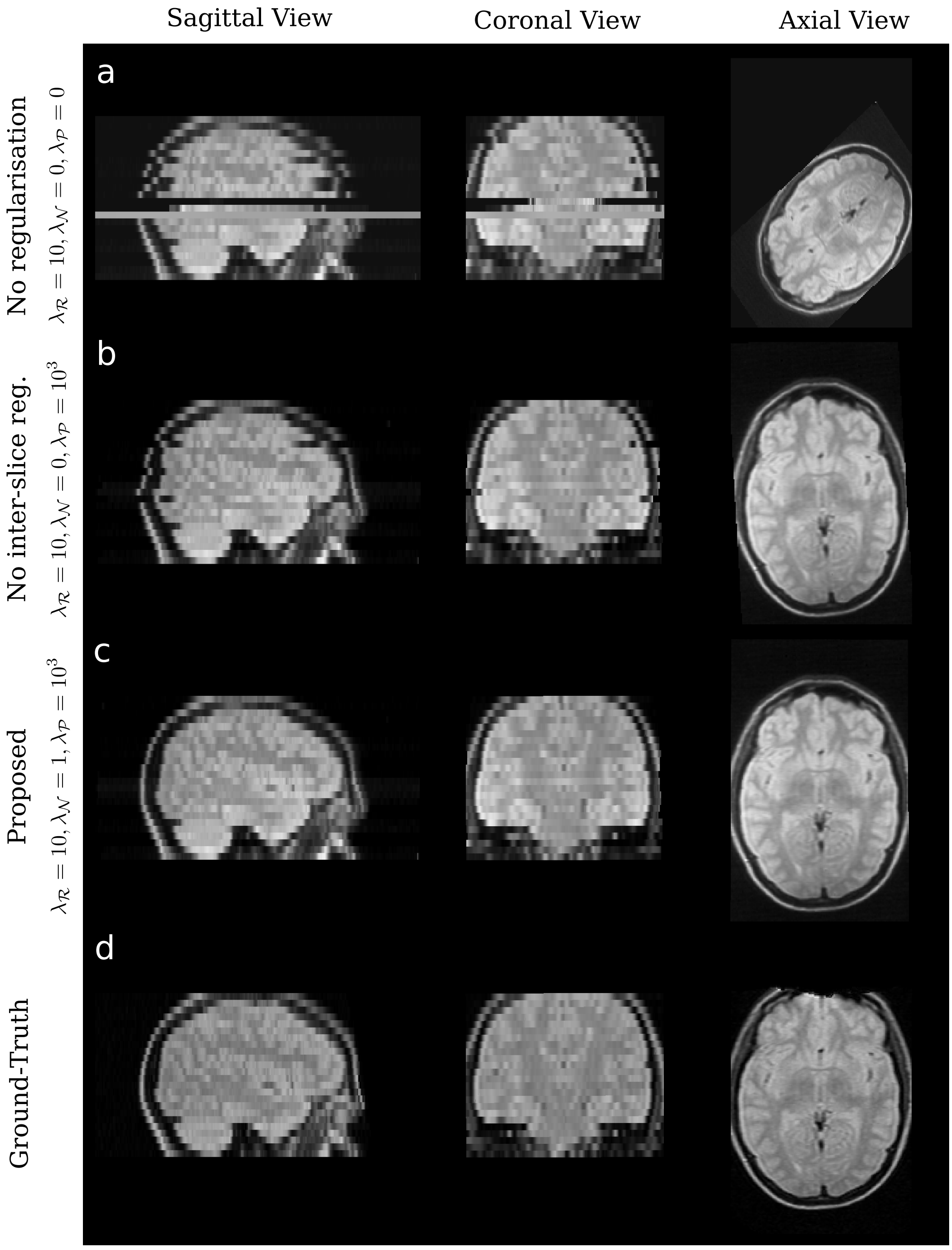

Figure S1: Reconstructed sample to show the importance of inter-slice regularisation for motion correction to obtain high-quality volumetric reconstructions. Severe misregistrations are visible in case motion correction is based on the reference image information alone (a). Adding the regularization prior term $\mathcal{P}$ achieves slight improvements (b). A volumetric reconstruction of high anatomical accuracy is achieved by using the proposed reconstruction pipeline based on the minimization problem (5) which additionally relies on inter-slice regularisation (c).
